# The neural basis of audiomotor entrainment: an ALE meta-analysis

**DOI:** 10.3389/fnhum.2014.00776

**Published:** 2014-09-30

**Authors:** Léa A. S. Chauvigné, Kevin M. Gitau, Steven Brown

**Affiliations:** NeuroArts Lab, Department of Psychology, Neuroscience & Behaviour, McMaster UniversityHamilton, ON, Canada

**Keywords:** entrainment, acoustic, finger tapping, cerebellum, basal ganglia, ALE, timing, meter

## Abstract

Synchronization of body movement to an acoustic rhythm is a major form of entrainment, such as occurs in dance. This is exemplified in experimental studies of finger tapping. Entrainment to a beat is contrasted with movement that is internally driven and is therefore self-paced. In order to examine brain areas important for entrainment to an acoustic beat, we meta-analyzed the functional neuroimaging literature on finger tapping (43 studies) using activation likelihood estimation (ALE) meta-analysis with a focus on the contrast between externally-paced and self-paced tapping. The results demonstrated a dissociation between two subcortical systems involved in timing, namely the cerebellum and the basal ganglia. Externally-paced tapping highlighted the importance of the spinocerebellum, most especially the vermis, which was not activated at all by self-paced tapping. In contrast, the basal ganglia, including the putamen and globus pallidus, were active during both types of tapping, but preferentially during self-paced tapping. These results suggest a central role for the spinocerebellum in audiomotor entrainment. We conclude with a theoretical discussion about the various forms of entrainment in humans and other animals.

## Introduction

The capacity of humans to synchronize movements to an external metric rhythm has attracted much interest in both evolutionary psychology (Patel, 2014) and experimental psychology (Repp, 2005). Such synchronization to an external rhythm is considered as a form of “entrainment.” However, the concept of entrainment is broader than that, as it applies not only to synchronization with external signals but also to interpersonal coordination (Knoblich and Sebanz, 2008; Schmidt et al., 2011; Phillips-Silver and Keller, 2012), such as when a rowing team rows in unison or when two people attempt to move a bulky sofa up a narrow staircase. Despite that, much recent discussion about entrainment has focused on the external type since it is phylogenetically rare, seen only in humans and a small number of other taxa (Merker et al., 2009; Patel et al., 2009; Schachner et al., 2009; Cook et al., 2013). For humans, this trait is expressed ubiquitously in dance across cultures, where people entrain their body movements to metric rhythms, such as drum beats (Jordania, 2006), where metric rhythms are temporal sequences in which accents appear regularly such that perception of predictable beats emerges (Kung et al., 2013). In addition, humans are quite prone to performing such synchronization in an unconscious manner (Repp and Keller, 2004), such as when they spontaneously tap body parts to the beat while listening to music. Given the fact that there is little compelling evidence that non-human primates can move to a beat (although see Zarco et al., 2009; Hattori et al., 2013), audiomotor entrainment—the ability of an animal to move in synchrony with an external beat—has been seen as a signature feature of human evolution, akin to bipedality (Larsson, 2014) and perhaps related to it through the evolution of dance. Hence, there has been a large interest in identifying the neural underpinnings of this sensorimotor trait in humans and in understanding what kind of brain changes may have underlain its evolutionary emergence.

Experimentally, the standard paradigm for looking at entrainment to an external beat is paced finger tapping (see Repp, 2005 and Repp and Su, 2013 for reviews), for which there is a substantial literature, both behavioral and neuroscientific. Given the fact that all rhythmic motor behaviors are driven by internal timekeeping mechanisms, the key question for external entrainment is how such motor-timing mechanisms—which are universal across animals and are thus generic—become synchronized with external oscillators like metronome beats to generate the highly specialized trait of audiomotor entrainment. Sensorimotor synchronization can be thought of in terms of the temporal coordination between internal motor-timing mechanisms and the timing of the perceived stimulus. One way that this can be examined is to compare acoustically-paced movements with self-paced movements having the same tempo, thereby comparing external and internal determinants of movement timing. A major objective of the present article is to perform a comparison between externally-paced and self-paced finger tapping studies in order to identify brain areas specifically associated with acoustic entrainment.

Neuroimaging studies of finger tapping have provided the major testing ground for neural models of both rhythmic motor production and sensorimotor entrainment in humans. While many components of the motor system are involved in rhythmic finger tapping (Witt et al., 2008), much of the discussion of motor timing has focused on two key subcortical networks, namely the cerebellum and basal ganglia. These two systems have both been proposed as the candidate timekeeper of the brain, where a timekeeper is an entity that keeps track of timing, either as a clock for duration-based timing or as a metronome for beat-based timing. While these two systems have been described in relation to general timing mechanisms (both perception and production), we will focus our attention here on studies of production, as related to the rhythmic finger tapping paradigm.

The cerebellum is considered as a central structure for the control of internal timing (Mayville et al., 2002; Ivry and Spencer, 2004; Jantzen et al., 2004; Bengtsson et al., 2005; Pecenka et al., 2013). The neocerebellum, which is the cerebellum's lateral division, is thought to process timing *per se* and to be one of the clock mechanisms of the brain (Kawashima et al., 2000; Schubotz et al., 2000; Mayville et al., 2002; Oullier et al., 2005; Thaut et al., 2008; Keren-Happuch et al., 2014). The spinocerebellum, its medial division, is considered to be more involved in sensorimotor processing, including motor timing (Jäncke et al., 2000; Brown et al., 2006; Kornysheva and Schubotz, 2011; De Guio et al., 2012). Ivry and Spencer (2004) proposed that the processing of time is distributed across the cerebellum and that different cerebellar regions are activated depending on the context in which the timing has to be processed.

The basal ganglia have received much attention as a brain network involved in timing, both perceptual and motor. Indeed, the basal ganglia have been proposed to act as an internal clock of the brain that generates internal timing representations, in part related to dopamine signaling from the substantia nigra (Mayville et al., 2002; Ivry and Spencer, 2004; Jantzen et al., 2004; Grahn and Brett, 2007; Coull and Nobre, 2008; Thaut et al., 2008; Teki et al., 2011; Hove et al., 2013; Kung et al., 2013). According to such a model, the putamen acts as a time accumulator, i.e., a coincidence detector between oscillatory firing and dopaminergic inputs (Mayville et al., 2002; Ivry and Spencer, 2004; Wiener et al., 2010; Teki et al., 2011; Hove et al., 2013). While some researchers believe that the basal ganglia are more involved in controlling motor behaviors, rather than being general timing structures (e.g., Boecker et al., 1998), others argue that they function as an internal clock that supports both perceptual timing and motor timing, thereby having the potential to function independent of motor processes (Mayville et al., 2002). As with the cerebellum, it is also possible that different basal ganglia structures have different timing-related functions.

Regarding rhythm, the basal ganglia are often considered as a beat-based timing system (Grahn and Brett, 2007; Teki et al., 2011; Hove et al., 2013; Kung et al., 2013), which encodes isochronous stimuli and supports the basic processing of regular and predictable timing (Thaut et al., 2008). The basal ganglia are involved both in generating an internal rhythm and in finding the beat of an external stimulus by detecting its temporal regularity (Teki et al., 2011; Kung et al., 2013). In contrast, the cerebellum performs more-complex timing processing, such as encoding polyrhythmic stimuli (Thaut et al., 2008), establishing the duration of discreet stimuli (Ivry and Spencer, 2004; Teki et al., 2011), or performing a correction of timing errors led by the basic processing in the basal ganglia (Teki et al., 2011; Kung et al., 2013). Therefore, according to some models, the basal ganglia perform basic timing processing and the cerebellum, through its reciprocal connections with the basal ganglia, performs subsequent timing adjustments or other complex timing processes (Rao et al., 2001; Thaut et al., 2008; Teki et al., 2011).

Given our interest in understanding not just timing *per se* but sensorimotor entrainment in particular, what is the activity of these networks in externally-paced vs. self-paced motor behaviors? The cerebellum and lateral premotor cortex (both ventral and dorsal parts) are the most common areas activated in externally-paced motor tasks. Indeed, the cerebellum plays a role in sensorimotor synchronization (Jahanshahi et al., 1995; Rao et al., 1997; Weeks et al., 2001), the lateral premotor cortex plays a role in movements guided by external sensory stimuli (Jahanshahi et al., 1995; Larsson et al., 1996; Rao et al., 1997; Jäncke et al., 2000; Kawashima et al., 2000; Weeks et al., 2001; Kornysheva and Schubotz, 2011; Pecenka et al., 2013), and the cerebellum and lateral premotor cortex are reciprocally connected (Jahanshahi et al., 1995; Rao et al., 1997). In addition, the caudal part of the supplementary motor area (SMA proper) is activated by the execution of externally-triggered sequences (Kawashima et al., 2000; Weeks et al., 2001; Lehéricy et al., 2006) and is thought to mediate a comparison between external rhythms and internal timing representations (Schubotz et al., 2000; Jantzen et al., 2007), On the other hand, a network from the putamen to the rostral part of the SMA (pre-SMA) is often highlighted in studies of self-paced tasks (Rao et al., 1997). Internally-guided movements and self-paced tasks often elicit activity in the basal ganglia, particularly the putamen (Jenkins et al., 2000; Cunnington et al., 2002; Garraux et al., 2005). Similarly, the SMA, and especially the pre-SMA, is often involved in monitoring motor timing and preparing for internally-guided sequences and self-triggered movements (Jahanshahi et al., 1995; Boecker et al., 1998; Jäncke et al., 2000; Jenkins et al., 2000; Kawashima et al., 2000; Cunnington et al., 2002; Mayville et al., 2002; De Guio et al., 2012).

However, the problem with any simple model of motor timing is that most of the abovementioned areas have been found in studies of *both* self-paced and externally-paced production. For example, Jenkins et al. (2000) found that both externally-paced and self-paced tasks elicited activity in the putamen, but that the self-paced task led to greater activation (see also Boecker et al., 1998). Similarly, both types of tasks are shown to activate the dorsal premotor cortex, sometimes to a greater extent for externally-triggered tasks (Larsson et al., 1996; Kawashima et al., 2000). In addition, Jantzen et al. (2004) showed that the network activated by self-paced tasks was dependent on the context in which the pace was determined. Indeed, many of the abovementioned self-paced studies did not distinguish whether the pace was purely self-determined or if it was determined by prior tempo instructions in the experiment. According to Jahanshahi et al. (1995), movements where the pace is indicated by prior instructions should not be called self-paced, even if they are done in the absence of an external stimulus. We will return to this important point below.

Of interest to our analysis is the small number of studies that have performed direct contrasts between externally-paced and purely self-paced rhythmic tasks. Kornysheva and Schubotz (2011) had subjects perform finger tapping either to the beat of an auditory rhythm or at a freely determined rate while listening to sounds devoid of rhythm (thereby controlling for auditory stimulation). The contrast of externally-paced with self-paced tapping showed activations in several regions, including bilateral auditory areas and the left lateral premotor cortex. In a separate session, subjects received transcranial magnetic stimulation (TMS) over the left ventral premotor cortex. Doing so led to a disruption of externally-paced tapping but not self-paced movements. An functional magnetic resonance imaging (fMRI) scan several minutes following TMS showed that stimulation of the ventral premotor cortex led to activity in the cerebellar vermis (lobule V). Vermal activation in this post-TMS fMRI scan was inversely correlated with the external-pacing impairment caused by TMS and thus reliably predicted how well subjects preserved audio-motor synchronization. It could therefore be related to a process of audio-motor timing correction (Kornysheva and Schubotz, 2011).

Brown et al. (2006) had dancer subjects perform patterned leg movements that were either externally-paced to music or self-paced at the same general tempo (in the absence of music). The contrast of acoustically-paced movement vs. self-paced movement revealed not only expected activations in the auditory cortex (since the self-paced condition lacked music) but activity in the anterior vermis (lobule III) of the spinocerebellum. The cerebellar activation was not driven by music *per se*, since subtraction of passive music listening did not reduce the z score of the vermal activation. Hence, the spinocerebellar activation reflected sensorimotor entrainment rather than sensory or motor processing alone. This entrainment-contrast further revealed activity in the medial geniculate nucleus of the thalamus, leading the authors to propose a “low road” model of acoustic entrainment in the spinocerebellum in which the auditory information driving entrainment comes to the cerebellum principally from ascending (subcortical) rather than descending (cortical) auditory pathways.

In the present study, we employed activation likelihood estimation (ALE) meta-analysis to a broad corpus of neuroimaging studies of finger tapping in order to examine brain areas involved in entrained vs. self-paced finger tapping. A previous ALE meta-analysis of finger tapping carried by Witt et al. (2008) set the stage for several of the findings reported in the current study. The major limitation of that study, from our standpoint, is that the authors did not perform an entrainment contrast; in other words, they did not examine the direct contrast between acoustically-paced and self-paced tapping, although they analyzed each condition separately and used logical images to demonstrate overlap in the activation patterns between the two. Despite this limitation, the study reported a number of important findings. The authors characterized the basic brain network involved in rhythmic motor production, including the primary sensorimotor cortex (SMC), SMA, basal ganglia, cerebellum, premotor cortex, and parietal cortex. They also compared acoustically-paced, visually-paced, and self-paced finger tapping. The ventral premotor cortex was shown to have a preference for acoustically-paced tapping, while the SMA was shown to be primarily activated by self-paced tapping, which is concordant with the literature described above. The basal ganglia and the thalamus were shown to be activated by both acoustically-paced and self-paced tapping.

One problem with their analysis relates to how they classified some of the studies. In particular, we feel that certain tapping studies defy a simple categorization into externally-paced or self-paced types. For example, there are studies of “continuation tapping” in which subjects initially tap in synchrony with an external timekeeper but then continue tapping at the same tempo in the absence of the external signal. In addition, there are studies in which subjects learn to tap at a particular pace during a training phase of the study and then tap on their own during an experimental phase. We can think of these two types of paradigms as being examples of “memory pacing” driven by auditory imagery of a previously-heard metric rhythm. For Witt et al. (2008), both of these types of protocols were included as part of their “no stimulus” condition and thereby combined with studies of true self-paced tapping. We find this to be problematic since such paradigms are contaminated by an external pacing signal, even if it not present at the time of tapping (Jahanshahi et al., 1995). Hence, one of our key objectives was to restrict ourselves to studies of true self-paced tapping when examining the contrast with acoustically-entrained tapping. Memory pacing became a third category of pacing in our analysis.

The principal objective of the current study was to use voxel-based meta-analysis techniques to identify the major brain areas involved in acoustic entrainment in order to better understand the evolution of this trait in humans. Along these lines, we examined the finger tapping literature with the aim of comparing studies of acoustically-paced and self-paced tapping. We used the relevant literature employed in the Witt et al. (2008) meta-analysis as our starting point and updated the analysis to the present year. Acoustically-paced tapping in these studies was done to the beat of either an isochronous stimulus (the majority of studies) or to a more complex rhythm. The use of both was justified since isochronous stimuli represent the simplest form of rhythm, where the beat is equivalent to the stimulus (or a multiple of it). That is, in both cases, the perception of the regularity of the rhythm allows the generation of an internal model, which predicts the upcoming beat. In addition, since Witt et al. (2008) included studies of memory tapping in their self-paced category, we wanted to rectify this situation by only using studies of true self-paced tapping as the comparison group for acoustically-entrained tapping in order to create the purest entrainment contrast. We ran a conjunction analysis of studies of acoustically-paced and self-paced tapping in order to identify regions commonly activated by both types of pacing. We also ran contrast analyses to identify brain areas preferentially activated by each type of pacing. We were particularly interested in differentiating the role of the cerebellum and basal ganglia in the two types of pacing. Finally, we analyzed studies of memory tapping separately in order to determine how, given their implicit pacing signals, their activation pattern compared with both externally-paced and self-paced tapping. We discuss these results in the broader context of a general model of entrainment types.

## Materials and methods

### Inclusion criteria

Meta-analyses of published neuroimaging studies of acoustically-paced and self-paced finger-tapping tasks were performed using ALE meta-analysis (Turkeltaub et al., 2002) in order to compare brain activations across these two types of pacing. Articles were initially obtained from a previous meta-analysis of finger tapping (Witt et al., 2008). Additional articles, published through March 2014, were retrieved by searching the Medline database using the PubMed search engine with the search terms “finger tapping fMRI,” “finger tapping positron emission tomography (PET),” and “self-paced tasks.” In order to identify papers that might have been missed, we performed a more thorough search of the Medline database using the OVID engine with a Boolean search paradigm. Finally, the reference sections of the resultant articles were searched for additional studies. A full listing of the studies included in the meta-analyses is found in Table 1.

**Table 1:** **Studies included in the meta-analyses**.

**Experiments**	**Subjects**	**Contrasts**	**Foci**	**Pacing type**	**Hand**	**Fingers**
Albouy et al., 2012	30	Tapping vs. rest, training	12	Self-paced	Left	Sequence
Aoki et al., 2005	10	Index finger vs. listen	1	Externally-paced	Right	Index
		Ring finger vs. listen	7	Externally-paced	Right	Ring
		Double finger vs. listen	12	Externally-paced	Right	Pairs
Aramaki et al., 2006	15	Parallel vs. listen	18	Externally-paced	Bimanual	Pair I-M
		Miror vs. listen	7	Externally-paced	Bimanual	Pair I-M
Bijsterbosch et al., 2011	16	Regular vs. rest	11	Externally-paced	Right	Index
		Subliminal vs. rest	16	Externally-paced	Right	Index
		Supraliminal vs. rest	10	Externally-paced	Right	Index
Blinkenberg et al., 1996	8	Finger tapping vs. rest	10	Externally-paced	Right	Index
Calautti et al., 2001, old group	7	RH tapping vs. listen	4	Externally-paced	Right	Index to thumb
		LH tapping vs. listen	10	Externally-paced	Left	Index to thumb
Calautti et al., 2001, young group	7	RH tapping vs. listen	10	Externally-paced	Right	Index to thumb
		LH tapping vs. listen	10	Externally-paced	Left	Index to thumb
Catalan et al., 1998, 1999	13	Sequence 12 vs. listen	9	Externally-paced	Right	Sequence
		Sequence 16 vs. listen, controls	12	Externally-paced	Right	Sequence
Chen et al., 2006	11	Finger tapping 0 dB vs. silence	7	Externally-paced	Right	Index
De Guio et al., 2012	10	Unpaced tapping vs. rest, children	30	Memory paced	Right	Index
Gerardin et al., 2000	8	Motor execution vs. listen	24	Externally-paced	Right or left	Index or pair I-L
Jantzen et al., 2005	12	Auditory Synchronize pacing vs. rest	7	Externally-paced	Right	Index to thumb
Jantzen et al., 2005	12	Auditory Synchronize continuation vs. rest	4	Memory paced	Right	Index to thumb
Joliot et al., 1998	5	Finger tapping vs. rest, PET	13	Self-paced	Right	Index
Joliot et al., 1999	8	Finger tapping vs. rest, PET	11	Self-paced	Right	Index
		Finger tapping vs. rest, fMRI average	16	Self-paced	Right	Index
		Finger tapping vs. rest, fMRI correlation	20	Self-paced	Right	Index
Kadota et al., 2010	10	Right hand tapping vs. rest	6	Self-paced	Right	Sequence
		Left hand tapping vs. rest	8	Self-paced	Left	Sequence
		Both hands tapping vs. rest	13	Self-paced	Bimanual	Sequence
Kawashima et al., 2000	8	Memory timed finger movement vs. rest	10	Memory paced	Right	Index
Kuhtz-Buschbeck et al., 2003	12	Motor execution simple RH vs. baseline	4	Externally-paced	Right	Index to thumb
		Motor execution simple LH vs. baseline	9	Externally-paced	Left	Index to thumb
		Motor execution complex RH vs. baseline	8	Externally-paced	Right	Sequence
		Motor execution complex LH vs. baseline	12	Externally-paced	Left	Sequence
Kung et al., 2013	11	Tap isochronous vs. silence	15	Externally-paced	Right	Index
Larsson et al., 1996	8	Self-paced movement vs. rest	12	Memory paced	Right	Index
Lehéricy et al., 2006	12	Simple vs. rest	8	Externally-paced	Right	Index
		Scale vs. rest	11	Externally-paced	Right	Sequence
		Complex vs. rest	27	Externally-paced	Right	Sequence
Lerner et al., 2004	10	Tapping vs. listen, controls	9	Externally-paced	Right	Index
Lissek et al., 2007	33	Simple non-DH vs. rest	14	Self-paced	Left	Index
		Simple DH vs. Rest	15	Self-paced	Right	Index
		Complex non-DH vs. rest	28	Self-paced	Left	Sequence
		Complex DH vs. rest	37	Self-paced	Right	Sequence
Matthys et al., 2009	18	Finger tapping vs. baseline, no mirror	13	Memory paced	Right	Index
Mayville et al., 2002	9	Motor only vs. rest	5	Memory paced	Right	Index to thumb
Mostofsky et al., 2006, control children	11	Right-handed finger sequencing vs. rest	3	Self-paced	Right	Sequence
		Left-handed finger sequencing	5	Self-paced	Left	Sequence
Nyberg et al., 2006, group1	8	Before, trained sequence vs. rest	4	Self-paced	Left	Sequence
		Before, untrained sequence vs. rest	4	Self-paced	Left	Sequence
		After, trained sequence vs. rest	2	Self-paced	Left	Sequence
		After, untrained sequence vs. rest	2	Self-paced	Left	Sequence
Nyberg et al., 2006, group2	8	Before, trained sequence vs. rest	4	Self-paced	Left	Sequence
		Before, untrained sequence vs. rest	4	Self-paced	Left	Sequence
		After, trained sequence vs. rest	2	Self-paced	Left	Sequence
		After, untrained sequence vs. rest	2	Self-paced	Left	Sequence
Oullier et al., 2005	15	Executed synchronization vs. rest	17	Externally-paced	Right	Index to thumb
Peres et al., 2011	15	Finger tapping vs. rest	19	Self-paced	Right	Index
Rao et al., 1997	13	Synchronization vs. rest (interval 300 ms)	4	Externally-paced	Right	Index
		Synchronization vs. rest (interval 600 ms)	3	Externally-paced	Right	Index
Rao et al., 1997	13	Continuation vs. rest (interval 300 ms)	7	Memory paced	Right	Index
		Continuation vs. rest (interval 600 ms)	7	Memory paced	Right	Index
Riecker et al., 2006, young group	10	Movement vs. listen, main effects	6	Externally-paced	Right	Index
Riecker et al., 2006, old group	10	Movement vs. listen, main effects	8	Externally-paced	Right	Index
Roessner et al., 2012, control children	16	Finger tapping vs. rest	27	Memory paced	Right	Index
Rounis et al., 2005	16	Main effect of movement vs. listen	17	Externally-paced	Right	Random finger
Sadato et al., 1997, experiment 1	12	Mirror vs. listen	13	Externally-paced	Bimanual	Sequence
		Parallel vs. listen	15	Externally-paced	Bimanual	Sequence
Sadato et al., 1997, experiment 2	9	Right unimanual vs. listen	3	Externally-paced	Right	Index
		Left unimanual vs. listen	6	Externally-paced	Left	Index
		Bimanual mirror vs. listen	12	Externally-paced	Bimanual	Index
		Bimanual parallel vs. listen	13	Externally-paced	Bimanual	Index
Thaut et al., 2008	12	Polyrhythmic movements vs. listen	26	Externally-paced	Right	Index
		Isorhythmic movements vs. listen	9	Externally-paced	Right	Index
Vuust et al., 2006	18	Tap vs. listen	8	Externally-paced	Right	Index
Weeks et al., 2001	22	Internal move vs. rest	9	Memory paced	Right or left	Index or middle
Wylie et al., 2013	18	Auditory-paced finger tapping vs. rest	5	Externally-paced	Right	Index
Yoo et al., 2005	10	Group-level finger tapping activation vs. rest	17	Externally-paced	Right	Sequence

*The studies are listed in alphabetical order by first author. Forty-three finger tapping experiments and 77 contrasts are included in the meta-analyses. The number of foci, the type of pacing, and the hand(s) and finger(s) used in the tasks are indicated for each contrast. In the Fingers column, “sequence” refers to an alternation between at least three fingers in a specific order, and “pair” refers to the tapping of two fingers simultaneously. Abbreviations: I, index finger; M, middle finger; L, little finger*.

Experimental conditions in which subjects performed finger tapping to an auditory pacing cue were classified as “externally-paced.” Experiments in which subjects performed tapping without external pacing were divided into two sub-categories based on whether or not a prior entrainment/training phase of the experiment specified a tapping rate to the subjects. Only conditions that lacked both external pacing and any prior indication of a tapping rate were classified as “self-paced.” Intermediate types of conditions, in which tapping occurred without acoustic pacing but was preceded by either a previous bout of entrainment (continuation tapping) or a training phase with a metronome, were classified as “memory-paced.”

Our inclusion criteria for the studies were: (i) that brain scanning was performed using either fMRI or PET; (ii) that papers reported activation foci in the form of standardized stereotaxic coordinates in either Talairach or Montreal Neurological Institute (MNI) space; (iii) that subjects were healthy individuals, thereby excluding studies using clinical populations but including studies of healthy children; (iv) that the pacing stimulus for the externally-paced conditions was auditory, thereby excluding studies of visual pacing of tapping; (v) that the analyses included contrasts against rest or a suitable low-level control condition; and (vi) that results from the entire scanned volume were reported, thereby excluding studies scanning only a portion of the brain or that only reported region-of-interest analyses. Note that studies that did not include data from minor parts of the brain, such as the most inferior part of the cerebellum, were included. Wherever studies reported multiple experiments from the same group of subjects, the contrasts were included together as a single study. For studies that reported the results of more than one subject-group, each group was treated separately, in accordance with the approach of Turkeltaub et al. (2012).

As a result, 43 studies were included in our meta-analysis, including 25 externally-paced tapping experiments (295 subjects, 469 foci), nine self-paced tapping experiments (128 subjects, 244 foci), and nine memory-paced tapping experiments (116 subjects, 124 foci). During all the experiments, participants tapped at an isochronous rate (including four self-paced experiments where they tapped as fast as possible). Among the externally-paced studies, 23 experiments used an isochronous auditory stimulus, whereas two used musical rhythms. For 24 of the externally-paced experiments, subjects' tapping was supposed to occur on every beat of the stimulus, whereas one study used hemiola tapping to an isochronous stimulus.

### Analysis

GingerALE 2.3 (www.brainmap.org/ale) was used for all analyses as well as for converting MNI coordinates into Talairach coordinates. The ALE results were registered onto a Talairach-normalized template brain using Mango 3.1 (ric.uthscsa.edu/mango). Separate meta-analyses were conducted for externally-paced (*n* = 25), self-paced (*n* = 9), and memory-paced (*n* = 9) tapping. All individual analyses were corrected for multiple comparisons using the false discovery rate (FDR) *p* < 0.01 with a cluster threshold of *k* = 120 mm^3^.

In addition to running individual analyses, we performed a conjunction analysis and direct statistical contrasts between the externally-paced and self-paced ALE maps (Nichols et al., 2005) in order to identify areas that were specific for acoustic entrainment. The conjunction was generated by taking the smallest ALE value between the two individual ALE maps (FDR corrected *p* < 0.01 for individual maps). The contrast analyses were performed at *p* < 0.05 uncorrected on the previously-corrected individual ALE maps, with a cluster threshold of *k* = 120 mm^3^. Note that visual comparison between the individual meta-analyses might lead to misleading conclusions due to the difference in the number of studies in each analysis. However, such a difference is corrected for statistically in the conjunction and contrast analyses (Eickhoff et al., 2011). The one caveat to point out is that the low number of self-paced studies may have produced a bias toward more variance in its ALE analysis, thereby resulting in false increases in the number or size of clusters in the contrast of self-paced vs. externally-paced tapping.

## Results

**Figure 1:**
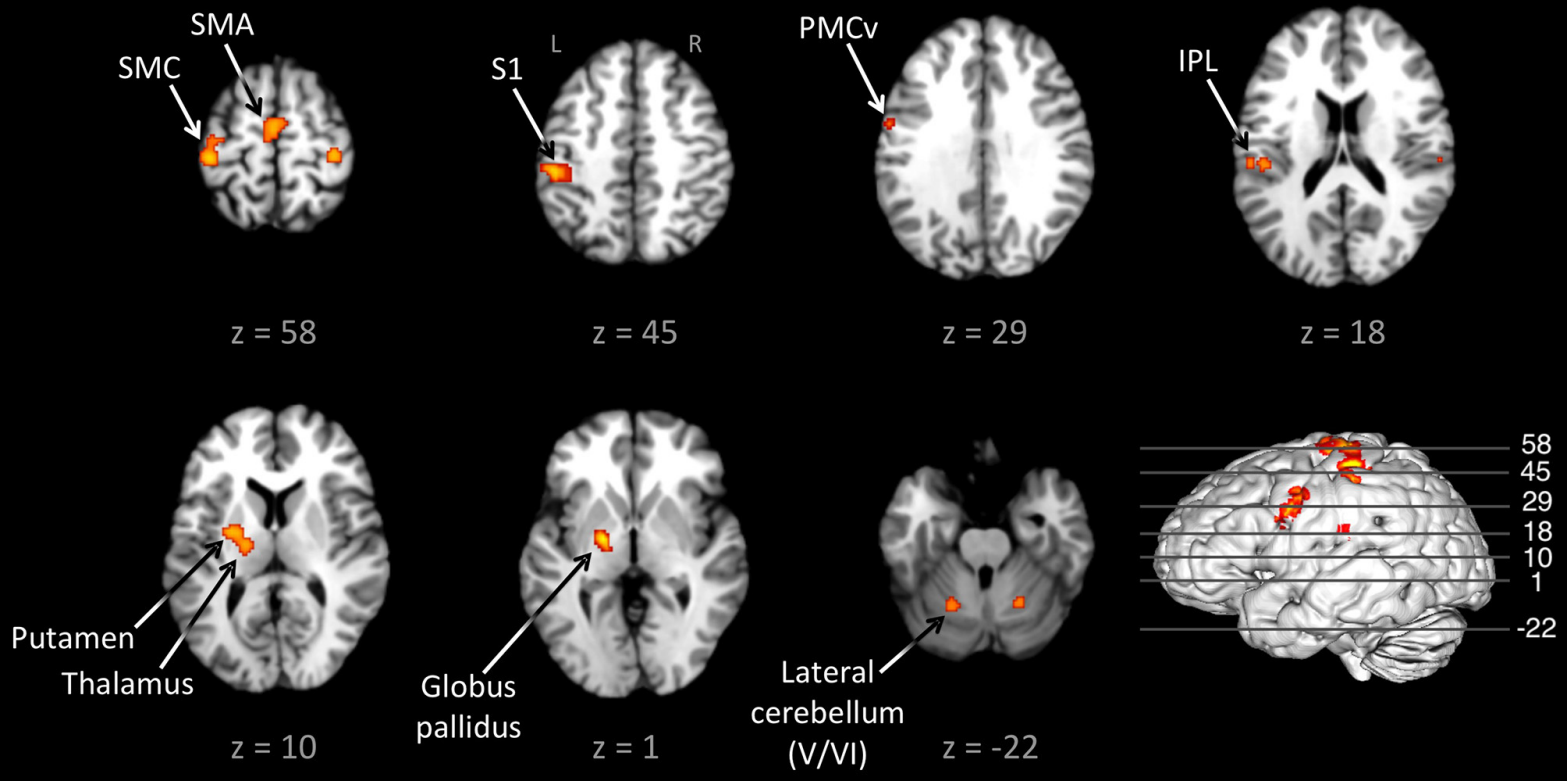
**Conjunction between the externally-paced and self-paced ALE maps**. The analysis is *p* < 0.01, FDR corrected. The 3D brain is shown to indicate the slice levels. The slices are shown in neurological convention. Abbreviations: IPL, inferior parietal lobule; L, left; PMCv, ventral part of the premotor cortex; R, right; S1, primary somatosensory cortex; SMA, supplementary motor area; SMC, sensorimotor cortex.

Figure 1 shows the conjunction of activations for externally-paced and self-paced tapping, demonstrating the common brain network underlying rhythmic finger tapping, irrespective of pacing type. Talairach coordinates for these ALE foci are presented in Table 2. Activations were seen in the bilateral SMC (somatotopic hand representation), caudal part of the SMA, left ventral and dorsal premotor cortex (BA 6), and bilateral inferior parietal lobule (IPL; BA 40). Regarding the two subcortical networks involved in timing, cerebellar activity was seen bilaterally in lobules V and VI, which is a region that includes the somatotopic finger representation of the lateral cerebellum (Grodd et al., 2001), itself linked to the bilateral activity seen in the SMC. Basal ganglia activity was seen in both the putamen and globus pallidus, but only in the left hemisphere. Activity was also seen in the nearby ventral lateral nucleus of the left thalamus, although this activity could not be unambiguously associated with either the cerebellum or basal ganglia alone.

**Table 2:** **Conjunctions and contrasts between the externally-paced and self-paced ALE maps**.

**Area**		***BA***	**Conjunction**				**External > self**				**Self > external**			
			***x***	***y***	***z***	***ALE***	***x***	***y***	***z***	**z score**	***x***	***y***	***z***	**z score**
**FRONTAL LOBE**														
M1	L	4	−34	−16	60	14.8								
	R	4	36	−24	56	18.5					30	−22	60	2.8
		4									38	−22	58	2.7
SMA	L	6	0	−4	54	21.7								
		6	−2	−10	58	17.8								
PMCd	L	6									−29	−11	64	3.5
PMCv	L	6	−56	0	30	15.9								
		6	−54	−4	36	14.4								
		6	−50	4	8	13.6								
**PARIETAL LOBE**														
S1	L	2	−48	−26	46	21.8								
		2	−52	−28	42	16.3								
		3	−38	−28	56	20.1								
	R	2									36	−38	60	2.3
		2									56	−24	36	2.3
		2									54	−24	32	2.1
		2									50	−20	26	2.1
		3									36	−34	60	2.3
IPL	L	40	−54	−24	20	15.5					−52	−32	48	2.8
		40	−46	−26	18	15.4					−50	−24	26	1.9
											−48	−22	22	1.8
	R	40	58	−22	20	14.2					44	−22	24	2.0
SPL	L	7									−26	−57	57	2.1
**TEMPORAL LOBE**														
Posterior STG	R	42					62	−24	8	2.0				
		22					58	−18	4	1.9				
**SUBCORTICAL**														
Putamen	L		−26	−4	12	18.6								
Globus pallidus	L		−18	−8	2	24.4					−14	−4	4	2.2
VL thalamus	L										−12	−10	4	2.3
**CEREBELLUM**														
Lateral (VI)	L		−20	−58	−20	15.1					−22	−60	−18	2.1
	R		20	−56	−22	14.3								
Lateral (V)	R						14	−52	−18	1.9				
Vermis (V)	L						10	−50	−24	1.9				
	R						15	−59	−13	2.1				
							10	−62	−16	2.1				
Vermis (VI/VII)	R						6	−60	−24	1.9				

*The Talairach coordinates of the significant ALE clusters are presented for the conjunction of externally-paced and self-paced tapping (p < 0.01, FDR corrected) as well as for the contrasts of External > Self and Self > External (p < 0.05, uncorrected). The ALE values for the conjunction represent the minimum ALE value from either the externally-paced or self-paced ALE maps. The ALE values shown are the true values times 10^‒3^. The statistical contrasts between the two ALE maps are given as z-scores for External > Self and Self > External. BA, Brodmann area; IPL, inferior parietal lobule; M1, primary motor cortex; PMCd, dorsal part of the premotor cortex; PMCv, ventral part of the premotor cortex; S1, primary somatosensory cortex; SMA, supplementary motor area; SPL, superior parietal lobule; STG, superior temporal gyrus; VL, ventral lateral nucleus of the thalamus*.

**Figure 2:**
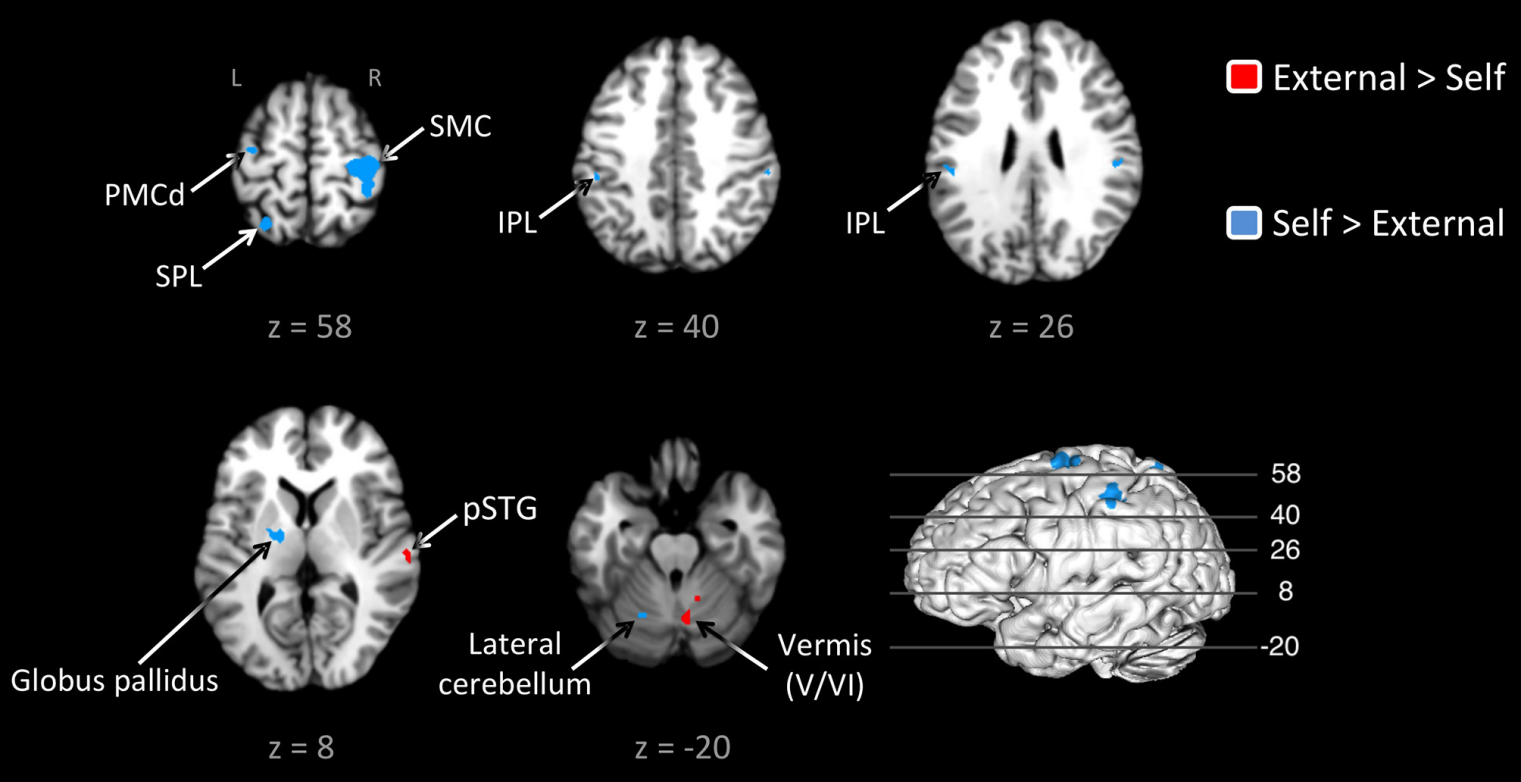
**Contrast analysis between the externally-paced and self-paced ALE maps**. The analyses are *p* < 0.05, uncorrected. The activations are color-coded according to the legend at the right. The 3D brain is shown to indicate the slice levels. The slices are shown in neurological convention. Note that the large activation in the right SMC and the corresponding activation in the left lateral cerebellum are simply a reflection of the larger number of left-handed studies for self-paced tapping (see text). Abbreviations: IPL, inferior parietal lobule; L, left; PMCd, dorsal part of the premotor cortex; R, right; SMC, sensorimotor cortex; SPL, superior parietal lobule.

Given this shared network, the next step was to perform reciprocal contrasts between the two types of pacing (Figure 2 and Table 2). The contrast of External > Self (red foci in Figure 2) revealed activity in only two regions. One was an expected activation in the auditory association cortex (posterior superior temporal gyrus, pSTG; BA 22), reflecting the exclusive presence of auditory stimulation in entrained tapping. The other area was the vermis of the cerebellum (lobules V and VI/VII) extending toward the right lateral cerebellum (lobule V). The reverse contrast of Self > External (blue foci) revealed a greater number of foci. Areas of activation included the right SMC, left dorsal premotor cortex (BA 6), left superior parietal lobule (BA 7), bilateral IPL (BA 40), and left lateral cerebellum (lobule VI). It is important to point out that the activity in the right SMC and left lateral cerebellum is mainly related to the larger number of left-handed studies in the self-paced analysis compared to the entrained analysis (see below), and may not be a reflection of neural differences between self-pacing and external pacing. Finally, activity was seen in the left basal ganglia, mainly in the globus pallidus and ventral lateral nucleus of the thalamus. Thus, these two subtractions revealed a double dissociation between the subcortical timing circuits: External > Self showed activity in the cerebellar vermis, while Self > External showed activity in the globus pallidus of the basal ganglia.

**Figure 3:**
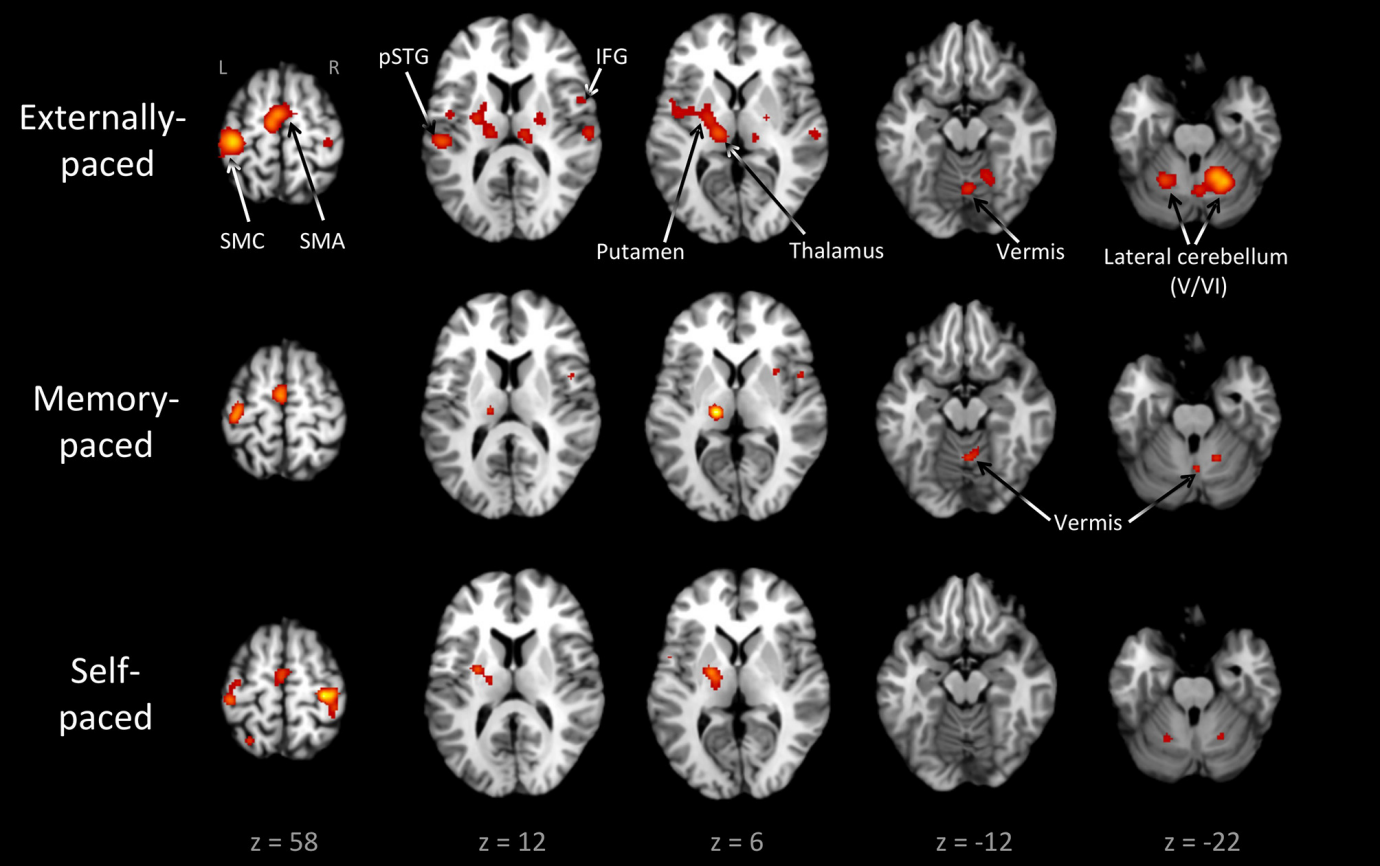
**Individual ALE maps for the three types of pacing studied**. The analyses are *p* < 0.01, FDR corrected. The slices are shown in neurological convention. Abbreviations: IFG, inferior frontal gyrus; L, left; pSTG, posterior superior temporal gyrus; R, right; SMA, supplementary motor area; SMC, sensorimotor cortex.

Figure 3 and Table 3 present the individual ALE analyses, including that for memory pacing. As mentioned in the Introduction, memory pacing is an intermediate case between external and self-pacing, since the tapping occurs in the absence of a pacing cue but is driven by auditory imagery of a previously heard cue. Memory-paced finger tapping showed activity in the same basic network described above for the conjunction of external and self-pacing, but also included areas that were seen in externally-paced tapping but not self-paced tapping, namely the cerebellar vermis, inferior frontal gyrus (IFG; BA 44), and ventral posteromedial nucleus of the thalamus. This activation profile suggests that memory pacing is much closer to entrained tapping than it is to self-paced tapping. Finally, regarding the subcortical timing circuits, it is important to point out that the left putamen was present for both external pacing and self-pacing, while the vermis was only present for external pacing and memory pacing, but not self-pacing.

**Table 3:** **The three types of pacing studied**.

**Area**		***BA***	**Externally-paced**				**Memory-paced**				**Self-paced**			
			***x***	***y***	***z***	***ALE***	***x***	***y***	***z***	***ALE***	***x***	***y***	***z***	***ALE***
**FRONTAL LOBE**														
M1	L	4	−38	−22	54	53.7	−36	−24	56	21.3				
	R	4	36	−24	56	18.5					30	−24	58	28.4
SMA	L	6	−6	−12	54	47.0	−2	−10	56	20.4	0	−4	54	21.7
		6	−2	−4	58	38.9	−6	−4	52	19.7	−2	−10	58	17.8
PMCd	L	6									−32	−12	62	18.2
PMCv	L	6	−54	0	28	17.7					−56	0	32	17.5
		6	−54	−6	34	17.0					−54	−4	38	14.4
		6									−52	4	8	14.0
IFG	R	44	56	4	20	22.3	48	8	8	13.2				
**PARIETAL LOBE**														
S1	L	3									−38	−28	56	20.1
	R	3	32	−30	52	17.3					38	−32	58	17.5
		3									54	−22	40	14.2
		2									54	−20	34	16.1
		2									58	−20	22	15.2
IPL	L	40	−52	−24	14	31.0	−46	−30	46	14.0	−48	−28	46	25.6
		40	−56	−28	36	30.1	−46	−28	26	13.6	−54	−24	20	15.5
		40	−46	−42	46	18.2					−54	−22	24	14.9
		40									−46	−26	18	15.4
	R	40	36	−40	42	22.3					46	−24	22	15.6
		40									56	−34	40	15.1
		40									36	−38	56	14.8
		40									52	−20	24	14.1
SPL	L	7									−26	−56	58	16.1
	R	5									36	−40	60	14.2
**TEMPORAL LOBE**														
Posterior STG	L	42	−44	−4	8	22.4								
	R	42	58	−20	10	23.8								
**SUBCORTICAL**														
Putamen	L		−24	−8	12	24.6					−26	−4	12	18.6
	R		22	−8	12	19.7	30	10	6	12.0				
Globus pallidus	L		−20	−8	2	29.0					−18	−8	2	24.4
Claustrum	L		−34	−2	4	19.3								
VPM thalamus	L		−14	−20	8	32.7	−16	−20	6	23.4				
	R		12	−22	10	24.2								
**CEREBELLUM**														
Lateral (V)	L		−22	−54	−24	30.8	16	−52	−20	13.9				
Lateral (VI)	L		−16	−52	−18	24.2					−20	−58	−20	15.1
	R		22	−54	−22	44.1					20	−56	−22	14.3
Vermis (IV)	R						8	−50	−14	14.5				
							2	−54	−12	13.7				
Vermis (V)	R		2	−62	−16	39.2								
Vermis (VI/VII)	R						2	−60	−22	13.0				
Dentate nucleus	R		16	−52	−20	47.7								

*The Talairach coordinates of the significant ALE clusters are presented for each of the three types of pacing in finger tapping tasks: externally-paced, memory-paced, and self-paced (p < 0.01, FDR corrected). The ALE values that are shown are the true values times 10^−3^. BA, Brodmann area; IFG, inferior frontal gyrus; IPL, inferior parietal lobule; M1, primary motor cortex; PMCd, dorsal part of the premotor cortex; PMCv, ventral part of the premotor cortex; S1, primary somatosensory cortex; SMA, supplementary motor-area; SPL, superior parietal lobule; STG, superior temporal gyrus; VPM, ventral posteromedial nucleus of the thalamus*.

In order to know if movement complexity had an influence on these results, we examined which fingers and hands were used across the different types of conditions. We found that 52% of the entrained experiments were done with only one finger (usually the index finger), compared to 33% of the self-paced experiments and 78% of the memory-paced experiments. We generated a “complexity” value for each experiment according to the number of fingers and hands used. The mean complexity value was 2.3, 4.0, and 1.4, respectively, for entrained, self-paced, and memory-paced tapping. A two-tailed unpaired *t*-test showed that the difference in complexity between entrained and self-paced tapping was not significant (*p* > 0.01).

Regarding the hand used in the tapping tasks, the left hand or both hands were used in 28, 66, and 11% of the experiments, respectively, for entrained, self-paced, and memory-paced tapping. These proportions explain the bilateral activations in the SMC and lateral cerebellum seen in the self-paced analysis, compared with the unilateral activations for both of these structures in the entrained and memory-paced analyses.

## Discussion

In this study, we sought to examine the neural basis of the phylogenetically rare ability of humans to entrain movements to a metric rhythm. To do so, we meta-analyzed the neuroimaging literature devoted to rhythmic finger tapping in order to identify regions of the brain specifically activated by externally-paced finger tapping, as compared with self-paced tapping. The results demonstrated a dissociation between the two major subcortical systems implicated in timing control. Contrast analyses revealed the importance of the spinocerebellar vermis for acoustically-paced tapping. The basal ganglia were observed for both types of pacing but were preferentially activated by self-paced tapping. Overall, these results suggest that entrained movement to the underlying beat of an acoustic rhythm, which is a novel human ability, is more related to the cerebellum than the basal ganglia, while the latter might be more important for internally-regulated control of movement timing (as well as for finding the underlying beat, see Kung et al., 2013). These results raise important evolutionary questions about acoustic entrainment, not least since the cerebellar vermis is a highly conserved structure among vertebrates and has even been shown to have undergone an evolutionary reduction in humans compared to non-humans primates (see below).

The conjunction analysis of externally-paced and self-paced tapping (Figure 1) replicated the basic findings of Witt et al. (2008), demonstrating a brain network for rhythmic finger tapping irrespective of pacing type, including the SMC, lateral premotor cortex, SMA, IPL, putamen/globus pallidus, and lateral cerebellum. This is not surprising given the strong overlap in the literatures covered by these meta-analyses. The major difference between our analysis and theirs was the stronger bilaterality of their profile, with bilateral activations in the IPL and basal ganglia that were both left-lateralized in our analysis. In addition, they observed activity in the IFG and pSTG that we only saw for externally- and memory-paced tapping. Table 3 reveals that this same basic network was activated across all three types of pacing examined in this study. Hence, this seems to be a general circuit for rhythmic control of finger movement.

Our interest in revisiting the finger tapping literature was not to look at metric motion *per se* but to identify brain areas specifically associated with entrainment. For that, it was necessary to employ the comparison task of self-paced tapping. While Witt et al. (2008) performed a comparison between auditory pacing, visual pacing, and “no stimulus” pacing, they only did so using logical analyses and not statistical contrasts. In addition, as mentioned in the Introduction, they included studies of memory pacing in their “no stimulus” category, hence contaminating the self-paced category with studies having an implicit external pacing signal. Indeed, it is possible that the brain regions responsible for entrainment maintain their activity even after the external stimulus is removed. Therefore, we wanted to perform a statistical contrast between entrained and self-paced tapping, with the additional provision that the self-paced corpus be free of the confounding effect of memory pacing.

The contrast of entrained vs. self-paced tapping revealed activity in the vermis of the spinocerebellum in addition to an expected activation in the auditory association cortex (pSTG). Examination of the individual ALE analyses showed that the vermis was present in the externally-paced analysis but absent in the self-paced analysis. Given that entrainment is typically viewed as a form of prediction, our results are consistent with the general role of the cerebellum in mediating prediction and in reducing prediction error during motor tasks (Tseng et al., 2007; Taylor et al., 2010; Kornysheva and Schubotz, 2011). One could argue that the activation in the vermis only reflects error correction between the stimulus and the tap during audio-motor synchronization, rather than entrainment *per se*. However, additional evidence for the role of the vermis in acoustic entrainment, rather than error correction, comes from the observation that the vermis was active during memory pacing, where no auditory stimulus was present. This result argues that the entrainment circuit of the spinocerebellum does not require external auditory input to stimulate it but that it can be driven by auditory imagery of a pacing signal, as processed by cortical auditory areas (Halpern and Zatorre, 1999). The IFG too could mediate the storage and rehearsal of auditory timing information (Rao et al., 1997). It plays a role in timed motor tasks whenever an auditory stimulus is involved, whether the stimulus is currently present or was previously presented (Kawashima et al., 2000; Bengtsson et al., 2005; Witt et al., 2008). Activation of the vermis and IFG during memory pacing suggests that this type of pacing is actually a form of covert acoustic entrainment.

Overall, the vermis, probably through its interaction with other motor-timing areas, emerged in this study as the strongest candidate for a brain area that mediates audiomotor entrainment, such as occurs not just in finger tapping (Jäncke et al., 2000; Kornysheva and Schubotz, 2011; De Guio et al., 2012) but in dance as well (Brown et al., 2006). In fact, our entrainment contrast replicated the results of the only motor-timing study performed using dancers. Brown et al. (2006) had tango dancers execute patterned leg movements that were either externally-paced to tango music or self-paced at the same general tempo. The contrast of acoustically-paced vs. self-paced movement revealed, beyond expected activations in the auditory cortex, activity in the anterior vermis of the spinocerebellum. Interestingly, the difference in the location of the vermal activation in the dance study (lobule III) and the finger-tapping meta-analysis (lobule V/VI) might reflect differences in the somatotopic location of the legs and fingers in the spinocerebellum. The classic map of the medial cerebellum shows an inverted homunculus in the anterior lobe such that the lower extremity is most anterior and the upper extremity is more posterior (Grodd et al., 2001).

The entrainment-contrast of Brown et al. (2006) further revealed activity in the medial geniculate nucleus of the thalamus. Based on this, the authors proposed a “low road” model of acoustic entrainment in the spinocerebellum in which the auditory information driving entrainment comes to the cerebellum principally from ascending (subcortical) rather than descending (cortical) auditory pathways. They argued that the beat information that drives entrainment—not least the unconscious kind of entrainment that routinely occurs when people listen to music—is coarsely-processed sensory information that does not require the elaborate spectral analysis that the auditory cortex is specialized at carrying out. However, the results of the present meta-analysis with memory pacing indicate that descending input from cortical areas involved in auditory memory can drive vermal activation. Interestingly, Petacchi et al.'s (2005) ALE meta-analysis of cerebellar activations during passive listening to acoustic stimuli did not reveal ALE foci in the vermis but only in more-lateral hemispheric regions, with the exception of Crus II posteriorly. Hence, vermal activation might be explicitly linked to *sensorimotor* processing, rather than sensory processing alone, as would be expected for an area that mediates entrainment. The finding that the vermis receives strong input from the primary motor cortex (Coffman et al., 2011) suggests that the vermis might be ideally situated to compare motor commands with ascending inputs from the sensory pathways in order to facilitate sensorimotor synchronization by reducing prediction error (see Kornysheva and Schubotz, 2011).

It is worth pointing out that the vermis is not the only part of the cerebellum that is implicated in timing. The bilateral cluster in the lateral cerebellum (lobule VI) observed in the conjunction analysis is close to the somatotopic finger representation (Grodd et al., 2001) but is also considered a key structure for generating internal timing representations (Kawashima et al., 2000; Schubotz et al., 2000; Mayville et al., 2002; Oullier et al., 2005; Thaut et al., 2008; Keren-Happuch et al., 2014). Several studies have found that timing complexity is linked with the lateral cerebellum, even when motor activity is controlled for Kawashima et al. (2000), Mayville et al. (2002), Oullier et al. (2005), Thaut et al. (2008). Lobule VI of the lateral cerebellum is consistently found in both perceptual and motor tasks involving timing, as shown by a meta-analysis of cerebellar function (Keren-Happuch et al., 2014). Therefore, the timing circuit of the cerebellum includes not only the vermis but the hemispheres as well. In contrast to the vermis, the lateral cerebellum does not show specificity for entrainment, as it was activated comparably by all three types of pacing (Figure 3).

What about the basal ganglia, the other major subcortical circuit strongly implicated in timing? The individual meta-analyses showed the basal ganglia to be active during both externally-paced and self-paced tapping. However, contrast analysis revealed that the globus pallidus was more active during self-paced compared with externally-paced tapping. The common presence of the basal ganglia in the externally-paced and self-paced analyses is consistent with the fact that both types of pacing are isochronous and that the basal ganglia are reliably activated by tasks that involve regularity and predictability, whether during perceptual, motor, or sensorimotor tasks. The basal ganglia, and especially the putamen, are involved in the processing of metric stimuli (Brown et al., 2006; Grahn and Brett, 2007) as well as in the generation and maintenance of internal representations of time (Jantzen et al., 2007; Coull and Nobre, 2008; Kung et al., 2013). Such representations can be purely self-determined without external cues (Mayville et al., 2002; Ivry and Spencer, 2004; Jantzen et al., 2007; Coull and Nobre, 2008; Hove et al., 2013) or they can be generated according to external stimuli (Jantzen et al., 2007; Coull and Nobre, 2008; Kung et al., 2013). Indeed, externally-paced movements that are regular and predictable can establish representations of movement timing that can be internally guided (Jahanshahi et al., 1995; Jäncke et al., 2000; Jenkins et al., 2000). Thus, the regular and predictable nature of isochronous tapping tasks elicits basal ganglia activity, as seen during both externally-paced and self-paced tapping. The residual activation of the basal ganglia for self-paced compared with externally-paced tapping is consistent with the well-known function of the basal ganglia in goal-directed (as opposed to stimulus-directed) movement and with movement initiation (Redgrave et al., 2010). Hence, the basal ganglia might play a stronger role in self-initiated movements than in movements entrained to external signals. The absence of the basal ganglia in the memory-pacing analysis (Figure 3) was unexpected. The detection by fMRI of activity in small internal structures such as the basal ganglia is less reliable than the detection of activity in cortical structures (Kawashima et al., 2000; Weeks et al., 2001; Yoo et al., 2005). The low number of foci in the memory-paced dataset compared to the externally- and self-paced datasets could also explain the absence of putamen activation.

The overall finding of the meta-analyses was a shared network of brain areas that was activated regardless of the pacing type, a network broadly supported by the literature reviewed in the Introduction. It includes not only frontal and parietal cortical areas (SMC, SMA, PMC, IPL) but the lateral cerebellum and putamen/globus pallidus. It also includes the ventral region of the thalamus that acts as a relay for both the cerebellum and basal ganglia in conveying information back to motor regions of the cortex (Asanuma et al., 1983; Haber and Calzavara, 2009). While the pallidal part of this shared network was shown to be more activated for self-paced tapping than externally-paced tapping, the vermis of the cerebellum was shown to be active for externally-paced tapping but absent for self-paced tapping, hence being a neural signature of entrainment. The vermis might be able to coordinate internal motor timing to the timing of external stimuli.

But this latter point raises an evolutionary conundrum. While the human capacity to keep the beat is a rarity among mammals, the vermis of the cerebellum is a highly conserved structure in vertebrates (Shmuelof and Krakauer, 2011). Even more paradoxically, Matano and Hirasaki (1997), in performing volumetric analyses of the cerebellum across 26 species of anthropoids, found that the targets of the vermis, namely the fastigial and interpositus nuclei, were reduced in volume (when controlling for the volume of the medulla) in humans compared to non-human primates, whereas the lateral cerebellum showed the reverse trend. This is certainly not the expectation that one would have for a specialized brain area that mediates a novel species-specific function. While we do not currently have a finite explanation for this, a prominent role might be played by the connectivity between the timing circuits of the basal ganglia (which support beat-based timing) and the cerebellum, where interactions have been proposed to occur in regions such as the pontine nuclei, inferior olive, and substantia nigra (Onodera and Hicks, 1998; Bostan and Strick, 2010; Teki et al., 2011). As beat-based timing is needed for both metrical self- and external-pacing, the brain network responsible for this timing seems to be necessary but not sufficient for audiomotor entrainment. In addition, non-human primates, as well other most other animals, lack the ability to find the beat. (Zarco et al., 2009; see also Merchant and Honing, 2014; Patel and Iversen, 2014). Thus, the capacity for entrainment could emerge from the connectivity between the cerebellar vermis and beat-based timing areas. Another important avenue to consider is the interaction between the medial and lateral zones of the cerebellum, possibly through their joint innervation by the primary motor cortex and through their complementary auditory input from ascending (medial cerebellum) and descending (lateral cerebellum) projections. Indeed, Zarco et al. (2009) showed that monkeys lack beat-based timing partly due to an inability to phase-adapt, a process needed during entrainment and which is supported essentially by the lateral cerebellum (Bijsterbosch et al., 2011). We therefore suggest that the vermis could be a central area in the entrainment network, responsible for synchronizing internal and external timing, and that others areas of the network, and connectivity between them, might have evolved to make acoustic entrainment a specific feature of humans. Further comparative research is necessary to address this important phylogenetic question about synchronization mechanisms.

### A classification of entrainment types

**Figure 4:**
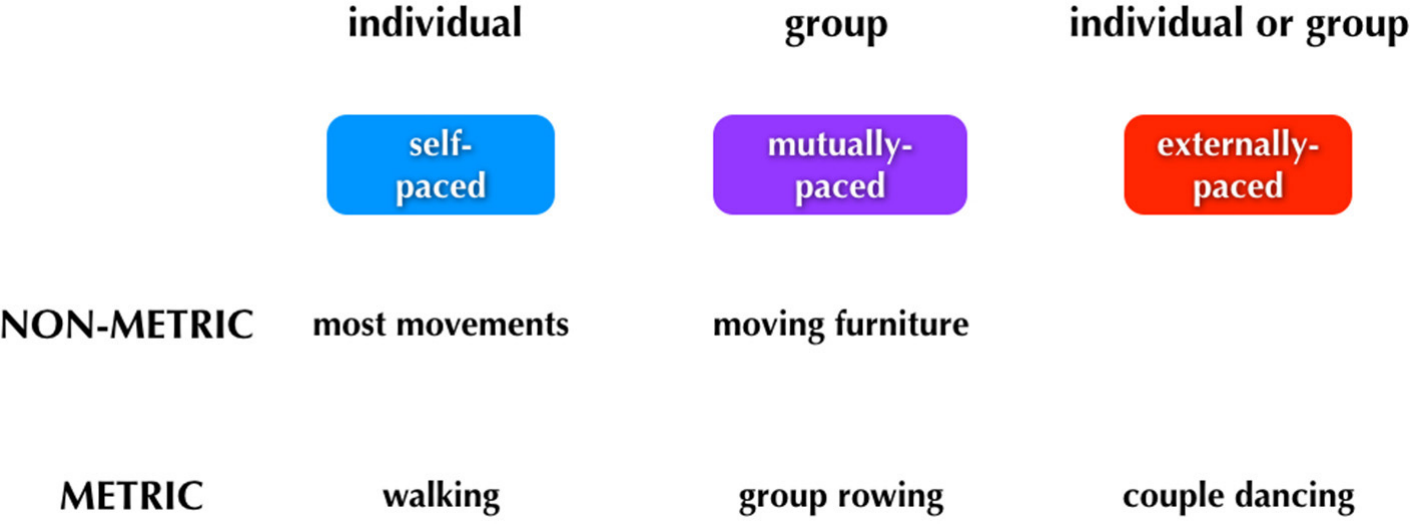
**Major categories of human pacing**. The figure summarizes the three major types of pacing. Examples of each type are shown below the boxes.

As mentioned in the Introduction, the concept of entrainment applies not only to synchronization with external signals but also to interpersonal coordination, such as when a rowing team rows in unison or when two people attempt to move a bulky sofa up a narrow staircase, situations where the tempo of movement is established mutually, not by some signal external to the group. As a conclusion to this article, we would like to present a framework for thinking about entrainment, one that covers all forms of human pacing (see Figure 4). In addition, we would like to highlight important differences between the pacing-types of music and dance (see **Figure 6**), since these two processes are often combined under the umbrella of “rhythmic” or “metric” behaviors.

We can think about three categories of timing: (1) *self-paced*, done only by individuals; (2) *mutually-paced*, done only by groups, and (3) *externally-paced*, done by either individuals or groups. The critical distinction between the latter two categories is whether the pacing-cue is coming from outside of the performers (external pacing) or whether it is negotiated internally by the group (mutual pacing). Distinguishing mutual pacing from external pacing might seem contradictory at first, since multiple individuals are influencing one another and thus acting as cues “external” to one another. However, if we focus on the group as a unit, then we can think of the tempo of the group's movements as being determined *internally to the group* rather than by some external pulse. Finally, rhythm is a component of the scheme. Whereas external pacing of movement is almost always done in a metric manner, self-pacing, and mutual pacing can be done in either a *non-metric* or *metric* manner.

Typical examples of movements of each type are shown below the boxes in Figure 4. Looking first to self-pacing, we see that the vast majority of movements carried out by individuals are self-paced and non-metric. There are important examples of self-paced movements that are done metrically, including walking and repetitive forms of work movements. It is worth noting that any kind of rehearsal without the presence of an external stimulus may reasonably involve imagery, such as the auditory imagery that could occur when a dancer is rehearsing without music. In this case, the movements would be memory-paced and not self-paced. Jumping now to external pacing, this occurs almost invariably in a metric manner. Dancing is a key example, whether done by an individual or group. Finally, mutual pacing can occur in either a non-metric or metric manner. An example of the former would be two people moving a heavy piece of furniture up a narrow staircase; such movement would be jerky and non-metric, although there might be short bouts of meter during it. An example of mutual pacing that is metric would be the movements of a rowing team. (Should there be a coxswain calling out the pace, then this would become a form of external pacing as well). As will be described below, the most complex aspect of the scheme relates to phenomena like group dancing to music in which external pacing and mutual pacing operate simultaneously. For example, the two individuals dancing a tango have to entrain *both* to a musical beat (external) and to one another (mutual), and this involves different sensory modalities and effector systems (see **Figure 6**).

**Figure 5:**
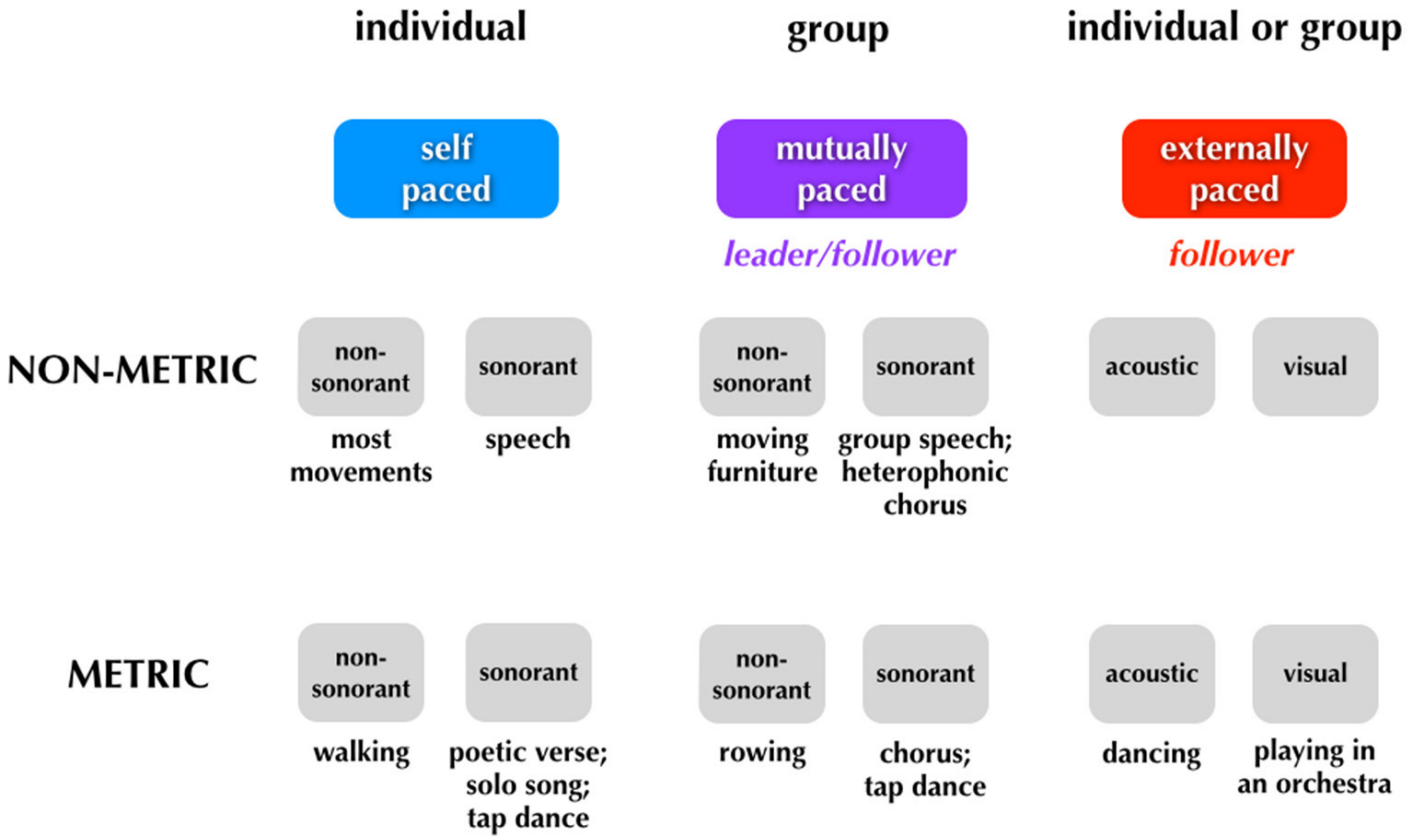
**Sonorant vs. non-sonorant movements**. This figure is similar to Figure 4 but adds new distinctions related to sonorance, leading/following, and the sensory modalities for external entrainment.

**Figure 6:**
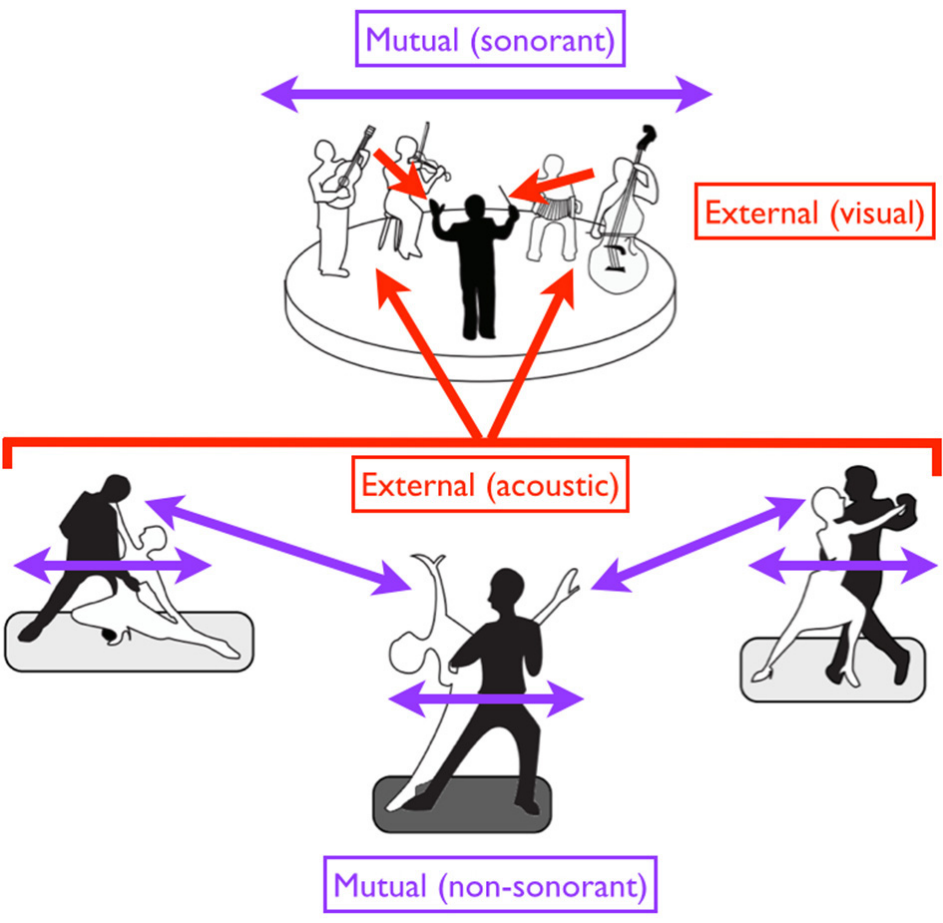
**A diversity of entrainment types in dancers and musicians**. Red arrows suggest external pacing, while purple arrows suggest mutual pacing. Black figures are leaders while white figures are followers. Regarding external pacing, the dancers are acoustically paced by the music, while the musicians are visually paced by the conductor. Mutual pacing is seen at two levels for the dancers: (1) within each couple (through both kinesthetic and visual interactions), and (2) between the “lead” couple in the center and the two outer couples (through visual interactions alone). Such pacing is non-sonorant. Mutual pacing is also seen at top among the four musicians of the ensemble, but this pacing is sonorant. In the case of mutual pacing, each individual or group of individuals can serve as both a leader and a follower, with the role alternating in an adaptive fashion. However, when individuals or groups are externally paced, they are purely followers.

Figure 5 presents the same scheme but adds some new distinctions to it as well as a few more examples of each category of movement. The first is a distinction between movements that are designed to be sound-generating (*sonorant* movements) vs. those that are not (*non-sonorant* movements). Whereas the vast majority of movements are non-sonorant (at least at the level of conscious awareness), sonorant movements occur during activities like speaking, singing, playing of a musical instrument, or the dancing that occurs while using body percussion, such as in tap dancing. The reason why sonorance is important in thinking about entrainment is that the sound self-generated by the movement creates cues for external entrainment. While all sensory cues have the potential to mediate entrainment, acoustic cues are far more effective (Repp and Penel, 2002, 2004; Witt et al., 2008). Thus, self-generated acoustic cues have a strong potential to influence entrainment (Phillips-Silver et al., 2010). This is seen routinely in group musical performance, where the sonorance of the production blurs the distinction between mutual and external pacing of movement, a problem that does not occur for non-sonorant movements (or even for sonorant though non-metric movements like speech). The second new distinction shown in Figure 5 relates to the idea that external pacing can occur using multiple types of sensory cues, including auditory and visual cues. Hence, whereas a tango couple is paced by the *acoustic* cues coming from an orchestra, the members of that orchestra are paced by the *visual* cues coming from a conductor (see also Figure 6). Mutual pacing as well employs multiple types of sensory cues, not just visual and auditory but also *kinesthetic* cues when there is physical contact between the members, as occurs very often in dance, but only rarely in music. Overall, any person interacting with other people in a joint activity is influenced by multiple timing cues such that their internal timing is moderated by both external and mutual pacing mechanisms.

Finally, the scheme in Figure 5 adds information about one more important component of pacing during group production, namely the distinction between *leading* and *following*. We usually think about this in the everyday sense of a tango couple in which one member of the pair is the leader (often the man) and the other member is the follower (often the woman). While we do not typically apply this distinction to solo movements, it seems reasonable to argue that any individual who is being paced by an external signal, for example recorded music, is a follower, whether in a solo context (e.g., a solo dancer) or a group context (e.g., a group of dancers). So, in Figure 5, external pacing is labeled as being an example of following. When dancers move to music, they generally do not have any ability to influence the tempo of the music and therefore do not have the ability to “lead” the music the way that the music leads them. The external signal acts as a leader. The most interesting and complex situation relates to mutual pacing in a group. We would argue that *any situation of mutual pacing by a group involves a leader-follower dynamic*. Moreover, this dynamic is fluid such that the roles can switch back and forth between members during the course of the movement. For example, when two people move a piece of furniture up a staircase, there might be times when the front person (the puller) is pacing the overall movement of the pair and other times when the back person (the pusher) is doing so. This is no less true during a duet between two musicians (Goebl and Palmer, 2009; Loehr et al., 2013; Palmer et al., 2013). The major point is that any situation of mutual entrainment requires a specification of a leader-follower dynamic. The greater the number of people that make up the group, the more complex (and potentially chaotic) the dynamic can become. Moreover, when group movement of this kind is paced by an external beat, such when a group of folk dancers moves to the beat of music, mutual pacing and external pacing interact.

This overall arrangement is summarized by the cartoon in Figure 6, in which we see three couples of tango dancers moving to music produced by a small ensemble, which itself is led by a conductor. Movement is non-sonorant in the case of the dancers but sonorant in the case of the musicians. Mutual pacing is seen (1) between the two members of each couple; (2) among the multiple couples; and (3) among the multiple musicians of the ensemble. Likewise, while a leader/follower distinction is seen within each couple, we can further imagine that a “lead couple” (shown by the middle couple in the figure) is serving as the leader of the other two couples. So, for the dancers, we have to consider both a between-couple and within-couple leader/follower arrangement for mutual pacing. External pacing is seen in both the dancers/musicians and musicians/conductor arrangements. Regarding sensory modalities for external pacing of movement, the dancers are being led by acoustic cues from the music, while the musicians are being led by visual cues from the conductor.

As mentioned above, when dancers move to the beat of music, they do not have any influence over the music and therefore do not have the ability to “lead” the music the way that it leads them. However, things are different when it comes to the mutual pacing between the two dance partners themselves. The hallmark feature of mutual pacing is *adaptivity*, the idea that members of the group can dynamically influence one another's movements and timing. Each member is both the sender and receiver of signals. Entrainment is *emergent*. Each member contributes to the generation of the pace, even if the leader has the more dominant role. This contrasts with external pacing, where producers are literally “following the beat”; in other words, they are pure followers. (It is important to keep in mind that during external pacing, the music that is serving as the “leader” for the dancers is itself produced by either a solo musician in a self-paced manner or a group of musicians in a mutually-paced manner through an interplay between leaders and followers). The literature on entrainment has focused almost exclusively on external pacing, most especially using the finger-tapping paradigm. Mutual pacing has been far less studied (though, see Phillips-Silver et al. (2010) for a theoretical model of mutual entrainment). A small number of finger tapping studies have looked at situations of “adaptive” tapping with virtual partners whose tempo varies over the course of a session (Repp and Keller, 2008; Fairhurst et al., 2013). What is strongly needed is a research program dealing with the nature of mutual pacing, including its leader/follower dynamic. Such a research program has to address the two problematic issues described above: (1) the relationship between mutual pacing and external pacing, such as when a dance couple moves to the beat of music or when a chorus performs with a conductor, and (2) the relationship between mutual pacing and external pacing when the movements are sonorant, such as when a chorus sings a cappella (Palmer et al., 2013).

Our final thought is about evolution. As mentioned in the Introduction, the human capacity for external entrainment has garnered much attention and has been analyzed by a large literature devoted to finger tapping. In reality, directed finger tapping of the type that occurs in a psychology experiment is one of the least naturalistic motor activities that people engage in; people are far more likely to tap their finger to music in an unconscious manner than they are to do so in a voluntary manner. The most naturalistic behavior that involves synchronization of movement to an acoustic beat is *dance*, either solo or in a group. So, the experimental finger-tapping paradigm is, in many respects, a model of dance, although it is never discussed as such. Likewise, the evolutionary analysis of the human capacity for acoustic entrainment is really an analysis of the evolution of dance. Finally, for all the discussion about external entrainment in animals (Patel et al., 2009; Schachner et al., 2009; Zarco et al., 2009; Cook et al., 2013; Hattori et al., 2013; Merchant and Honing, 2014), it needs to be pointed out that *mutual entrainment is the dominant—and most ancient—form of entrainment in the animal world*. Examples abound in the form of group locomotor behaviors (e.g., birds flying in formation, fish swimming in formation) and all forms of chorusing. This is especially expressed in non-metric forms. It is likely that the capacity for external entrainment is phylogenetically recent, having evolved from the capacity for mutual entrainment. While it might be the case that few animals are able to “follow the beat” when it comes to human-generated stimuli, we cannot allow this observation to obscure the fact that entrainment occurs on a massive scale in the animal world. Group locomotor and vocal behaviors are no less valid a topic for the analysis of entrainment than is a cockatoo bobbing its head to the beat of pop music. What is needed is an expansion of the research program on entrainment to include mutual pacing in humans and other animals.

### Conflict of interest statement

The authors declare that the research was conducted in the absence of any commercial or financial relationships that could be construed as a potential conflict of interest.
